# Evaluation of calcium hydroxide mixed with propolis by ultrasonic activation as root canal dressing in delayed tooth replantation

**DOI:** 10.4317/jced.61036

**Published:** 2024-03-01

**Authors:** Melyna Marques-de Almeida, Kevin-Luiz Lopes-Delphino, Vanessa-Fernanda da Silva, Francisley-Ávila Souza, Osvaldo Magro-Filho, Idelmo-Rangel Garcia-Júnior

**Affiliations:** 1 Health Sciences Center, Dentistry School of Jacarezinho, UENP – State University of Northern Paraná, Jacarezinho, Paraná, Brazil; 2 Department of Surgery and Integrated Clinic, Dentistry School of Araçatuba, UNESP - São Paulo State University. Araçatuba, São Paulo, Brazil

## Abstract

**Background:**

In cases of tooth avulsion, in which the neurovascular bundle responsible for nourishing the dental pulp is break, endodontic treatment is necessary before proceeding with tooth replantation. In this process, various substances have been tested in combination with calcium hydroxide Ca(OH)2 in an attempt to improve its effectiveness. This study aimed to examine the effects of using a mixture of Ca(OH)2 and 10% propolis, with subsequent application of ultrasonic treatment, on the delayed replantation of teeth in rats.

**Material and Methods:**

Twenty-four rats underwent a surgical procedure to extract the upper right incisor, leaving it on a surface to dry for one hour. The pulp and periodontal ligament were removed and the teeth were submerged in a 2% sodium fluoride acidulated phosphate solution. The canals were dehydrated using paper cones and the teeth were divided into four groups, according to the type of intracanal dressing: Ca(OH)2 group, Ca(OH)2 group with ultrasonic agitation, Ca(OH)2 and propolis group, Ca(OH)2 and propolis group with ultrasonic agitation. The root canals were irrigated with saline solution and the teeth were reimplanted. Sixty days after reimplantation, the animals were euthanized.

**Results:**

With regard to the presence of acute and chronic inflammatory infiltrate in the periodontal ligament, there was no statistically significant difference among some of the groups. Root resorption was identified in all groups, and there was no significant difference between them.

**Conclusions:**

It is concluded that the application of intracanal dressing containing Ca(OH)2 associated with 10% propolis, followed by ultrasonic agitation, did not prove to be more effective than the use of Ca(OH)2 alone in the repair process in the delayed replantation of rat teeth.

** Key words:**Tooth replantation, Calcium hydroxide, Propolis, Ultrasound, Intracanal dressing.

## Introduction

Dentoalveolar traumas represent a relevant problem because of their frequency, government expenditures and difficulty in prevention (1,2). The etiology is related to the different age incidence: in children, the main reason are a fall, sports, and aggression; while, after the age of 18, bicycle accidents, motorcycles, motorcycles, alcohol and drugs are more frequent (2,3).

The literature has shown that, among dentoalveolar traumas, dental avulsion occurs with a significant frequency in young patients due to bone resilience and also to incomplete rhizogenesis (3,4). The dental avulsion is defined as the complete displacement of the tooth from the interior of its alveolus causing, consequently, the rupture of the periodontal ligament and the vascular-nervous bundle that feeds the pulp (5), leaving the root surface exposed to the environment (1). Therefore, factors such as extra-alveolar period, storage medium and tooth contamination are crucial for the prognosis of the reimplanted tooth (1).

The viability of the periodontal ligament cells is the main factor influencing the repair of the periodontal ligament; this viability preserves the tooth itself from the external root resorption process (1). However, in the clinical everyday life, most of the re-implantations still occur late due to factors such as: lack of awareness of the population, involvement of professional health in the first care and even because the dentists themselves (6,7). Reimplantation should take place before periodontal ligament degeneration, which can be caused by long extra-alveolar time or inadequate storage media (1,8). This excessive extra-alveolar time leads to cellular necrosis and possible contamination of the root area, representing the main etiological factor of inflammatory resorption, which, although it is the only one that has treatment, is the most aggressive, and can lead to rapid tooth loss (1,10). In late permanent teeth reimplantation, different protocols have been well described for the control of this contamination, consisting in the treatment of the root surface (mechanical removal of the periodontal ligament and topical application of fluoride), administration of systemic antibiotic therapy and endodontic treatment (1,8).

Endodontic treatment should be performed before reimplantation when possible, or in a period of 7 to 10 days after late reimplantation (8). Treatment initiation timing is also related to the onset of inflammatory resorption, thus interfering with the prognosis of the lesion (9). The substances applied as intracanal medication aim to favor microorganisms elimination, to act as a physical-chemical barrier against infections or reinfections originated by salivary flora, to reduce periradicular inflammation, to solubilize organic material, to neutralize toxic products, to control persistent exudation and to stimulate repair by mineralized tissue (10).

The most used endodontic medication for this purpose is the calcium hydroxide paste (11). Hydroxyl ions and calcium ions present in this paste are capable of inactivating enzymes involved in the mechanism of nutrition of bacteria or acting directly on the cytoplasmic membrane, improving antimicrobial and tissue repair properties. In addition to activating tissue enzymes, such as alkaline phosphatase, they influence the mineralization and root resorption process (12,13).

Although its antimicrobial activity after direct contact with bacteria is almost immediate (12), its in-depth action within the dentinal tubules and possible ramifications of the main root canal has been observed in a longer period (14,15). Some microorganisms such as Enterococcus faecallis, the main responsible of endodontic failure, showed resistance to its action, and this could represent a limitation, (16,17).

Some drugs like paramonoclorophenol, chlorhexidine (18,19) and propolis (20,21) have been associated with Ca(OH)2 in order to increase its antimicrobial effect. The propolis is a resinous substance made by bees, during the collection of plant resins and enzymes from the saliva itself. Its chemical composition is considered complex because of the components present in it, and such substances change according to their geographical area (22). These variations influence their pharmacological properties. A great interest in the propolis of South American origin has been recently shown, due to the richness of the local flora. Samples of propolis varieties from Brazil and Venezuela showed high values of flavonoid compounds, phenolic acids being common (23).

Concerning its biological properties, the most prominent is antimicrobial, which acts mainly on Gram-positive bacteria and with limited activity against Gram-negative bacteria (24). There is evidence that the propole inhibites bacterial growth by preventing cell division, causing partial bacterial lysis and inhibiting protein synthesis (25). It has also shown anti-inflammatory, analgesic, anesthetic, antiulcer, immunostimulatory, hypotensive and cytostatic activity, besides being a potent flavonoid agent (26,27). Its antiinflammatory property is due to the capability of inhibition of prostaglandin synthesis and activation of the immune system, promoting phagocytosis and increasing healing effects on epithelial tissues. It has also been observed how it contains also iron and zinc, both important for the synthesis of collagen (28).

In addition, when compared to calcium hydroxide, propolis showed less toxicity to the periodontal ligament and pulp, being considered an intracanal drug alternative to calcium hydroxide (28,29). Decontamination is required not only in the root canal, but also in the root canal system (5). The mechanism of antimicrobial action of calcium hydroxide occurs through alkalinization of the dentin, so the drug infiltrates the dentinal tubules and root canal branches (13). The greater the depth of penetration and the greater the ion release the better the sanitation of the dentinal tubules (30).

In order to increase this diffusion of the drug in the dentin, ultrasonic agitation has been investigated. Ultrasonic agitation allows greater penetration of the dressing of delay in the tubules, increasing the ion release and, thus, occurring consequently greater alkalinization of the medium and greater decontamination (21,31). The calcium hydroxide paste associated to the propolis as a delay dressing used by ultrasonic agitation after late dental reimplantation could bring some benefit in the control of inflammatory root resorption, justifying the study. The objective of the study was to evaluate the effect of the use of intracanal calcium hydroxide dressing associated with the propolis at 10% followed by ultrasonic agitation in late reimplantation of rat teeth (5).

## Material and Methods

The research proposal and all experimental procedures were approved by the Ethics in Animal Research Committee of the School of Dentistry Araçatuba, UNESP, Brazil (Process No. 2013 - 01629). Thirty-two healthy adult (10-week-old) male rats (Rattus norvegicus Albinus, Wistar) weighing 180-200g were used. The animals were fed ground solid ration and water *ad libitum*, except for the preoperative 12h.

Before the procedures, the animals received an intramuscular injection of xylazine chlorhydrate (Dopaser; Laboratório Calier do Brasil Ltda. Osasco, SP, Brazil; 6mg per kg body weight) for muscular relaxation and were anesthetized with ketamine chlorhydrate (Dopalen; Agri Brands do Brasil Ltda.; 70 mg per kg body weight). Asepsis of the anterior maxilla was performed with polyvinylpyrrolidone-iodine followed by non-traumatic extraction of the maxillary right incisor of all animals. The teeth were bench dried at room temperature for 60 min. After the extra-alveolar time, the dental papilla and enamel organ were removed with a #15 scalpel blade and the pulp tissue was extirpated through the apical foramen with a slightly curved #35 Hedström file (Sybron Kerr Corporation, Glendora, CA, USA). Root canals were irrigated with saline (Ariston Ind. Quím. e Farm. Ltda, São Paulo, SP, Brazil) followed by aspiration. The root surface-adhered cemental PDL was removed with a #15 scalpel blade and the teeth were immersed in 20mL of a 2% acidulated-phosphate sodium fluoride solution, pH5.5 (0.1 M phosphoric acid, pH 2.0 diluted in 2% sodium fluoride solution, pH 8.0 (Apothicário Farmácia de Manipulação, Araçatuba, SP, Brazil) for 10 min. After root surface treatment, the canals were dried with absorbent paper points (Dentsply Ind. e Com. Ltda., Petrópolis, RJ, Brazil).

Four groups of teeth (n =8) were formed at random, according to the intracanal dressing.

Group Ca(OH)2 - the canals were filled with a paste prepared with Ca(OH)2 P.A. (Biodinâmica Quim. and Farm. Ltd, Ibiporã, PR, Brazil) and propyleneglycol (Laboratório Synth, Diadema, SP, Brazil) (5). Paste was prepared by mixing equal parts of powder and vehicle.

Group Ca(OH)2 with ultrasonic agitation - being manipulated and inserted in the same manner as described in the Group Ca(OH)2 (5). The Ultrasonic agitation was performed using the Multisonic-S Satelec Ultrasonic System (Gnatus Equipamentos Medicos-Odontológicos Ltda, Ribeirão Preto, SP, Brazil) with an introduction of a plain insert UA-05 (CVDentus, São José dos Campos), coupled without Satelec NSK DMC adapter (Gnatus Equipamentos Medicos-Odontológicos Ltda, Ribeirão Preto, SP, Brazil) (5). The orientation of the ultrasonic agitation in the mesio-distal and vestibular-lingual directions, being the execution time of 30 seconds in each direction. In order not to favor any specific region, during the execution time the operator performed penetration and removal movement in the direction of the long axis of the dental element, besides being careful not to touch the dentinal walls.

Group Ca(OH)2 and propolis - the canals were filled with a paste prepared with Ca(OH)2 P.A. (Biodinâmica Quim. and Farm. Ltd, Ibiporã, PR, Brazil) and solution of 10% propolis (Apothicário Farmácia de Manipulação). Paste was prepared by mixing in a ratio of 3:1 (powder / liquid) by weight. The 10% propolis solution was obtained from a 22 mL sample of ethanolic propolis extract (Unimel - Curitiba PR). The solution was taken in a water bath, extracting 2.4 g of propolis, which was then diluted in propylene glycol until the desired concentration was obtained (Aphoticário, Manipulation Pharmacy, Araçatuba-SP).

Group Ca(OH)2 and propolis with ultrasonic agitation - being manipulated and inserted in the same manner as described in the Group Ca(OH)2 and propolis. And received ultrasonic shaking as described in the Group Ca(OH)2 with ultrasonic agitation.

In each root canal, the paste was delivered through a retrograde route using a disposable insulin syringe. The paste was delivered up to the limit of 4 mm short of the apex and this region was filled with mineral trioxide aggregate (MTA, Angelus, Londrina, PR, Brazil). The sockets were gently irrigated with sterile saline and the teeth were replanted.

All animals received a single intramuscular dose of benzathine G penicillin 20,000 IU (Eurofarma Laboratórios Ltda., São Paulo, SP, Brazil). The rats were euthanized by anesthetic overdose 60 days after replantation. The teeth and surrounding tissues were surgically removed, fixed in 10% formalin for 24 h, decalcified in a 10% EDTA solution, pH 7.0 (Polyorganic Tecnologia Ltda, Campo Belo, SP, Brazil) and embedded in paraffin. Semi-serial longitudinal 6-µm-thick sections were obtained and stained with hematoxylin and eosin (H&E) for histomorphometric analysis.

A 4-point (1-4) scoring system was used to quantify the occurrence following histomorphometric events: site of epithelial reattachment, intensity and extent of the acute and chronic inflammatory process at the site of epithelial reattachment, PDL organization, intensity and extent of the of the acute and chronic inflammatory process in the PDL space, root resorption (active or inactive, extent, depth and repair), bone tissue, ankylosis. Data were analyzed statistically by the Kruskal-Wallis test followed by Dunn’s test for pair wise comparisons at a significance level of 5%.

## Results

**Figure 1:**
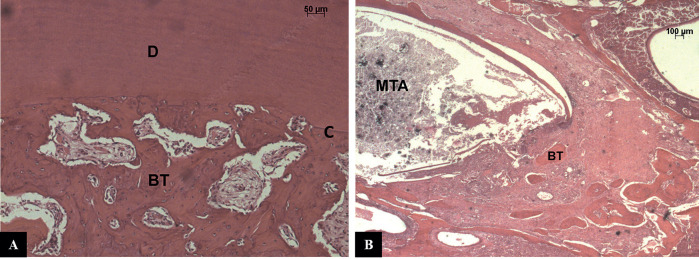
A - CH group – The periodontal ligament space was narrowed and filled by connective and bone tissues, and small areas of external root resorption were observed on dentin and cementum. Replacement resorption and some areas of ankylosis were evident along the root surface. Dentin (D), Cementum (C), Bone Tissue (BT). Hematoxylin and eosin (HE). 1. B - CH group: Connective tissue with presence of a chronic inflammatory infiltrate and new bone formation could be observed in contact with the apical MTA plug. Bone Tissue (BT), Mineral Trioxide Aggregate (MTA). Hematoxylin and eosin (HE).

Group Ca(OH)2 - In 4 of 6 specimens, epithelial reattachment to occurred at the cementoenamel (CEJ) (5). The underlying connective tissue had an inflammatory infiltrate that extended to near the alveolar ridge in the specimens where fracture of the alveolar ridge occurred (5). The periodontal ligament space was filled by connective tissue, the fibers of which were parallel to the root surface. The acute inflammatory process was present in all specimens, and in 4/6 specimens with large numbers of cells. Cement and dentin were present with extensive and deep areas of active resorption in all specimens. Small spots of ankylosis were observed along the root surface. In the apical region, near the biomaterial, there is presence of bone neoformation (Fig. 1A).

Group Ca(OH)2 with ultrasonic agitation - In 5 of 6 specimens, the epithelial insert occurred near the cement-enamel junction (5/6). The underlying connective tissue had an inflammatory infiltrate extending to the alveolar crest. The periodontal ligament space was filled by connective tissue whose fibers were arranged parallel to the root surface. In some areas, the bone tissue filled the entire periodontal ligament space. The acute inflammatory process was present in all specimens in the apical or coronary region of the root. Cement and dentin were present with extensive and deep areas of active resorption in all specimens. Small spots of ankylosis were observed along the root surface. In the apical region, close to the obturator material, bone neoformation is present (Fig. 1B).

**Figure 2:**
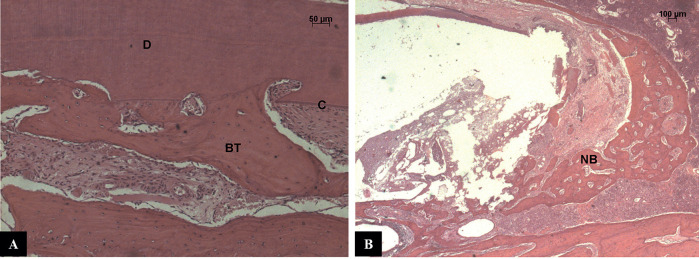
A - CH+CPMC (Calcium hydroxide + Paramonoclorophenol) group - Cementum and dentin presented areas of replacement resorption and ankylosis. Dentin (D), Cementum (C), Bone Tissue (BT). Hematoxylin and eosin (HE). 2. B - CH+CPMC (Calcium hydroxide + Paramonoclorophenol) group - In the apical region, it could be seen new bone formation and the connective tissue in contact with MTA presented an inflammatory infiltrate of mild intensity and limited extent. Mineral Trioxide Aggregate (MTA), New Bone (NB). Hematoxylin and eosin (HE).

Group Ca(OH)2 and propolis - In only one specimen, the reinsertion occurred slightly below the cement-enamel junction. The underlying connective tissue had an inflammatory infiltrate restricted to the lamina propria of the inner epithelium in most specimens. The periodontal ligament space was filled by connective tissue whose fibers were arranged parallel to the root surface and, in some areas, completely filled by bone tissue. The acute inflammatory process was present in all specimens in the apical or coronary region of the root, but with a small number of inflammatory cells. Cement and dentin were present with extensive and deep areas of active resorption in all specimens. Small spots of ankylosis were observed along the root surface. In the apical region, close to the obturator material, bone neoformation is present (Fig. 2A).

Group Ca(OH)2 and propolis with ultrasonic agitation - In 5 of 6 specimens, the epithelial insert occurred near the cementoenamel junction. The underlying connective tissue had an inflammatory infiltrate restricted to the lamina propria of the inner epithelium in 5 of 6 specimens. The periodontal ligament space was filled by connective tissue whose fibers were arranged parallel to the root surface and, in some areas, filled with bone tissue. The acute inflammatory process was present in all specimens in the apical or coronary region of the root, but with a small number of inflammatory cells. Cement and dentin were present with extensive and deep areas of active resorption in all specimens. Small spots of ankylosis were observed along the root surface. In the apical region, close to the obturator material, bone neoformation is present (Fig. 2B).

-Statistical Analysis

The statistical analysis showed a significant difference between the groups Group Ca(OH)2 and Group Ca(OH)2 and propolis compared with the groups Group Ca(OH)2 with ultrasonic agitation and Group Ca(OH)2 and propolis with ultrasonic agitation regarding intensity and extent of the acute inflammatory infiltrate in the periodontal ligament (Table 1). Also showed a significant difference between the groups Group Ca(OH)2 with ultrasonic agitation and Group Ca(OH)2 and propolis regarding extent of the chronic inflammatory infiltrate in the periodontal ligament.

**Table 1:**
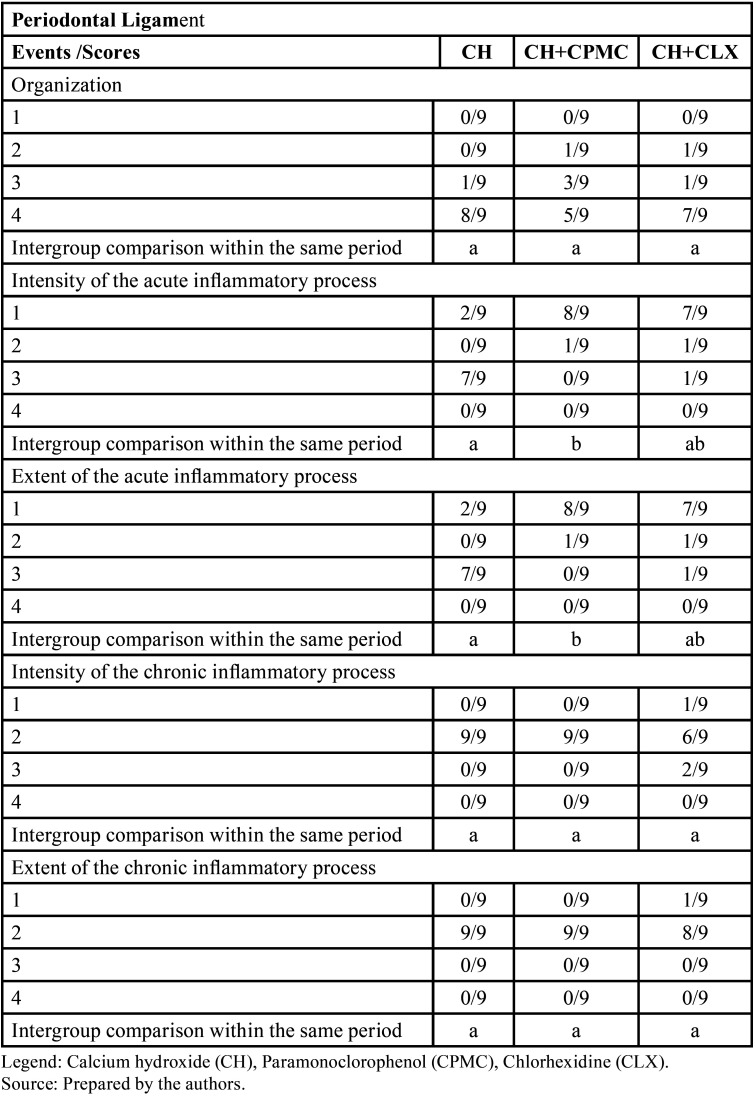
Scores and statistical analysis of the histomorphometric parameters in the periodontal ligament after Dunn’s test at 5% significance level.

## Discussion

The ideal treatment of dental avulsion is the dental reimplantation, when possible, even under unfavorable conditions regarding the viability of the periodontal ligament (1). When reimplantation can be performed only in a late time, when necrosis of the periodontal ligament has already occurred, the priority becomes to control the means of contamination, mainly by the root canal, in order to prevent external inflammatory root resorption, which leads to premature tooth loss (10).

The main accesses of contamination are: gingival, periodontal and pulp. Thus, to control these routes of contamination are indicated: root surface treatment, endodontic treatment and systemic antibiotic therapy (8,10,32). Root surface treatment has also been used as one procedure to limit root resorption. It consists in the mechanical removal of the periodontal ligament, followed by immersion of the teeth in acidulated phosphate sodium fluoride solution (8) in addition to the use of systemic antibiotic therapy (32), according to IADT protocol (8).

The control of contamination represents still one of the most evaluated topics in the endodontic literature. Calcium hydroxide is the most widely used drug for this purpose (11), and its mechanism of action is well known and has been described both *in vitro* (33) and *in vivo* (34). The high pH of the calcium hydroxide provides an inadequate environment for the survival of the bacteria inside the root canal, as well as for the reabsorption cells (12,13), and may favor repair after the elimination of the pathogens. Another important property (5) is that, in addition to eliminate the microorganisms, it degrades its endotoxins and exotoxins that sustain the toxic effect (35).

The vehicle used in the calcium hydroxide paste is a factor that influences the release of the calcium and hydroxyl ions during time. Water-soluble vehicles promote a faster release (36), explaining the choice of propylene glycol as a carrier of the pastes. In reimplantation it is very important that this alkalinizing action of the root canal and the dentinal tubules occurs as soon as possible because the installation of inflammatory resorption has already been observed histologically after 15 days of reimplantation (1). Various substances have been associated with calcium hydroxide in order to increase its antimicrobial power (19) and, among them, the propolis (21). Although its chemical composition depends on the species of bee and ecosystem (37), the propolis showed always as its inherent characteristic the antimicrobial action (38).

Some studies made use of ecosystem propolis originated by species of specific bees, which was extracted in the laboratories specifically for the research (21,24). However, the use of propolis associated with propylene glycol (obtained from an ethanolic propolis extract, easily found in the market) is also justified to allow the clinician’s access to an emergency, such as trauma cases.

The literature has shown that propolis presents little toxicity to the periodontal ligament and pulp when compared to calcium hydroxide, and may be suggested as an intracanal drug alternative to calcium hydroxide (28,39). Rezende *et al*. (40) observed, in an *in vitro* study, the association of calcium hydroxide with propolis and concluded that it was effective against dental infections, representing an alternative intracanal dressing, since it combines the characteristics of both compounds in order to obtain a beneficial biological effect.

Moreover, this association can spread through the dentinal tubules (20) and present a biocompatible effect with the tissues (28,39,40). One of the ways to improve this penetration of the drug in the dentin is through ultrasonic agitation, promoting better sanitation (21,31). Comparing the groups in which ultrasonic agitation was used, no statistically significant difference was observed in relation to the intensity of the acute inflammatory infiltrate in the periodontal ligament, suggesting that ultrasonic agitation leads to a greater penetration of the paste into the dentinal tubules, a greater alkalinization of the medium that ends up compensating the probable superior antimicrobial action of the mixture with the propolis reported in the literature (21).

This divergence from the literature may also be due to the endodontic treatment performed one hour after the avulsion, when the pulp had suffered an infarction and if it was found degenerate, but not yet in a state of decomposition that would favor a greater contamination both of the root canal and of the dentinal tubules, differently from teeth with pulp necrosis or *in vitro* work with previous dentin contamination (21).

However, when the chronic inflammatory infiltrate was observed, especially with respect to clastic cells, it was more intense in the propolis group without agitation when compared to Ca(OH)2 with agitation. Another reason could also be the higher alkalinization of the dentin (31), which interferes with the acute inflammatory process, which is related to the contamination, as well as the chronic one that, once controlled the contamination, due to the loss of the periodontal ligament, is characterized by the presence of external root resorption by substitution (1).

It was observed in previous studies (19) that the calcium hydroxide paste underwent a solubilization in the region of the apex that compromised its action. This experimental model as well showed a broad apical foramen simulating a tooth with incomplete rhizogenesis. Therefore, an apical plug with MTA was performed after the placement of the calcium hydroxide dressing, to avoid the direct contact of the paste with the periapical tissue, maintaining the action of the dressing only inside the root canal.

This technique of apical sealing with MTA was effective in preserving the action of the calcium hydroxide paste, maintaining the dressing inside the root canal and promoting bone neoformation in the apical region, confirming its biocompatibility in all groups. Root resorption was present in all groups without significant statistical difference (5), as usually expected when there is loss of the periodontal ligament (1,32). Although *in vitro* studies have reported satisfactory results with the association of Ca(OH)2 with propolis as a delay dressing in relation to its antimicrobial action (21,40), in late dental reimplantation this association was not superior to Ca(OH)2, probably because the contamination condition of the root canal and the dentinal tubules was different, since the pulp was removed one hour after the avulsion.

## Conclusions

It was concluded that the use of intracanal calcium hydroxide dressing associated with the 10% propolis followed by ultrasonic agitation was not more effective than Ca(OH)2, in the late rat reimplantation repair process.
